# Root Rot and Branch Dieback in *Arbutus unedo* Caused by *Phytophthora cinnamomi* in a Natural Woodland Area and Commercial Nurseries in Italy

**DOI:** 10.3390/life16101702

**Published:** 2026-10-11

**Authors:** Alessandra Benigno, Viola Papini, Federico La Spada, Mario Riolo, Domenico Rizzo, Santa Olga Cacciola, Salvatore Moricca

**Affiliations:** 1 Department of Agriculture, Food, Environment and Forestry (DAGRI), Plant Pathology and Entomology Section, University of Florence, Piazzale Delle Cascine 28, 50144 Florence, Italy; viola.papini@unifi.it (V.P.); salvatore.moricca@unifi.it (S.M.); 2 Department of Agriculture, Food and Environment, University of Catania, 95123 Catania, Italy; federico.laspada@unict.it (F.L.S.); mario.riolo@unict.it (M.R.); olgacacciola@unict.it (S.O.C.); 3 Laboratory of Phytopathological Diagnostics and Molecular Biology, Plant Protection Service of Tuscany, Via Ciliegiole 99, 51100 Pistoia, Italy; domenico.rizzo@regione.toscana.it

**Keywords:** strawberry tree, *Phytophthora cinnamomi*, oomycete pathogens, Mediterranean maquis, nursery trade, pathogen emergence

## Abstract

*Arbutus unedo* L. is an ecologically and economically important Mediterranean evergreen shrub or small tree, increasingly used in restoration and ornamental plantings. Despite its significance, its diseases and associated pathogens remain poorly studied. *Phytophthora cinnamomi*, one of the world’s most destructive plant pathogens, represents an emerging threat to Mediterranean ecosystems but has not previously been reported on *A. unedo* in Italy. In recent years, dieback has been observed in natural populations near Monticiano, Tuscany, while similar symptoms, associated with extensive mortality, have occurred in commercial nurseries in the Pistoia district. Woodland plants showed reduced vigour and hook-shaped shoot tips, whereas nursery plants exhibited root rot, collar necrosis, leaf chlorosis, crown dieback, wilting, and death. A total of 184 oomycete isolates were consistently recovered from symptomatic roots and collars and from rhizosphere soil. Morphological characterization and multilocus molecular analyses identified the isolates as *P. cinnamomi*. The pathogenicity of the oomycete was demonstrated by artificially inoculating healthy *A. unedo* seedlings, which displayed symptoms very similar to those observed under field conditions. Both representative isolates caused severe disease, resulting in 100% mortality of inoculated plants within 30 days, whereas no mortality occurred in control plants. Re-isolation of the pathogen from inoculated tissues fulfilled Koch’s postulates. These findings constitute the first report of *P. cinnamomi* on *A. unedo* in Italy and raise concern about its possible spread in natural ecosystems and through nursery production and trade.

## 1. Introduction

*Arbutus unedo* L. (Ericaceae), commonly known as the strawberry tree, is an evergreen woody species with a predominantly shrubby habit, although it may occasionally develop into a small tree [1]. It generally reaches 1.5–3 m in height, but under favourable environmental conditions individuals may attain 9–12 m [2,3]. The species is naturally distributed throughout the Mediterranean Basin, occurring across western, central and southern Europe, north-western Africa, western Asia, the Canary Islands, and extending along the Atlantic coast to south-western Ireland [4,5,6,7].

Within the Mediterranean Basin, *A. unedo* is typically associated with sclerophyllous shrublands and evergreen oak forests, where it is a characteristic component of the maquis vegetation and forest understory. It preferentially grows on siliceous or decarbonated substrates but can also tolerate moderately acidic to slightly alkaline soils, reflecting its remarkable ecological plasticity [3,6,8].

**Figure 1:**
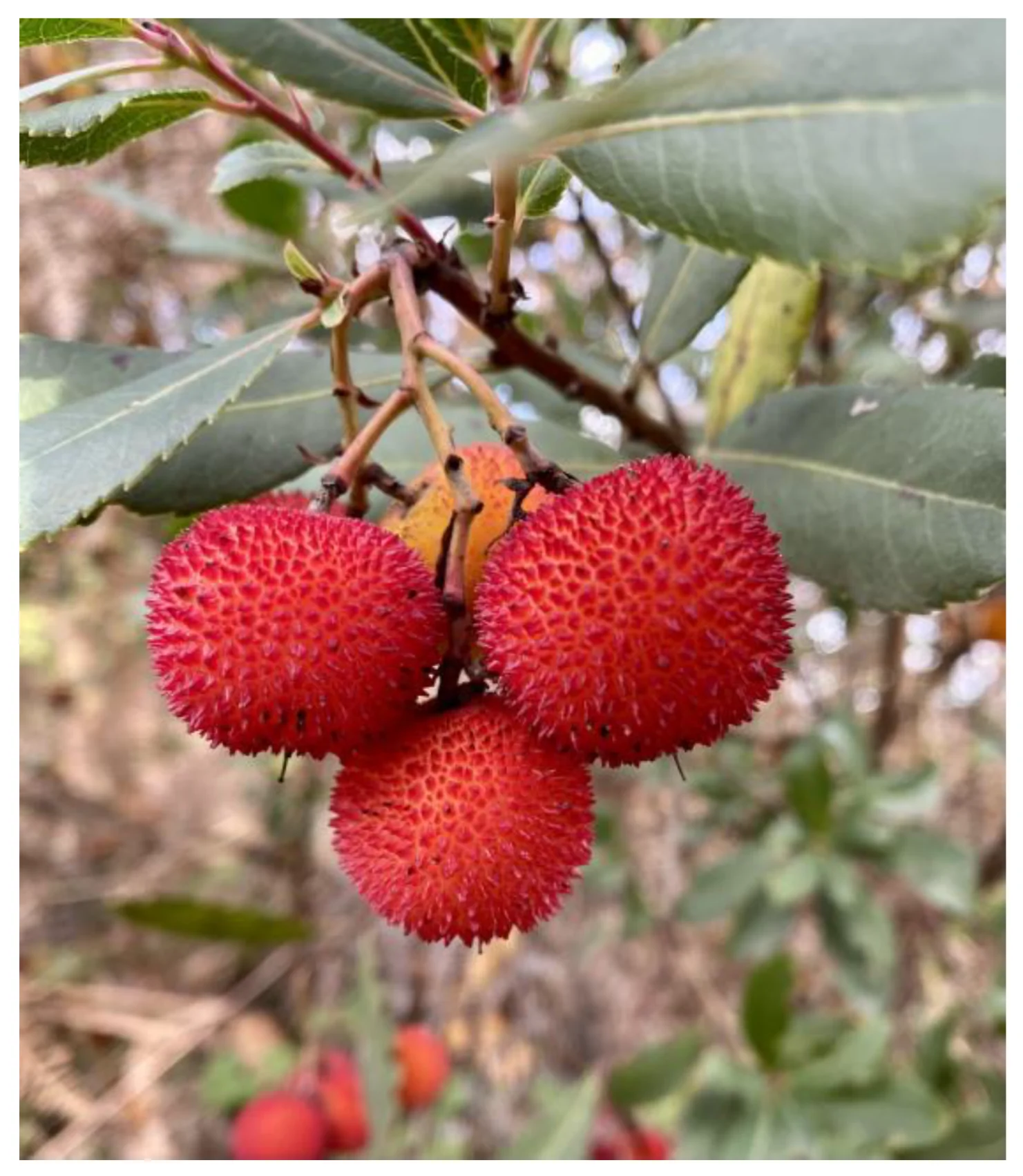
A close-up of a branch of *A. unedo* bearing fruit in late autumn. The ripe, globular, fleshy berries are covered in tiny warts. Their intense scarlet colour creates a striking contrast with the evergreen foliage.

*A. unedo* supports Mediterranean ecosystems through soil stabilization, erosion control and vigorous post-fire resprouting [9,10]. Its autumn–early winter flowers provide nectar and pollen for pollinating insects when other floral resources are scarce, while its fruits feed birds and small mammals [4]. These traits enhance biodiversity and ecosystem services, alongside its adaptability to urban environments. Its genetic, morphological and phenological variability reflects adaptation to diverse environmental conditions [11,12,13,14,15]. Apart from its ecological value, *A. unedo* is one of the most highly prized Mediterranean species for ornamental purposes, thanks to its aesthetic appeal. In fact, this species has acquired increasing economic relevance due to its ornamental value, edible fruits, medicinal properties and suitability for ecological restoration and reforestation programmes in Mediterranean landscapes [4,12,16]. The most distinctive feature of *A. unedo*, which makes it a highly prized plant for aesthetic and ornamental purposes, is the simultaneous presence of flowers and fruit—a relatively rare characteristic amongst European shrub species. The autumn flowering produces numerous white or cream-coloured flowers, arranged in pendulous panicles and characterised by an urceolate corolla typical of the Ericaceae family [2]. At the same time, the fruits resulting from the previous year’s flowering ripen: globose, fleshy berries covered with tiny protuberances (warts), which turn a vivid scarlet red when ripe (Figure 1).

**Figure 2:**
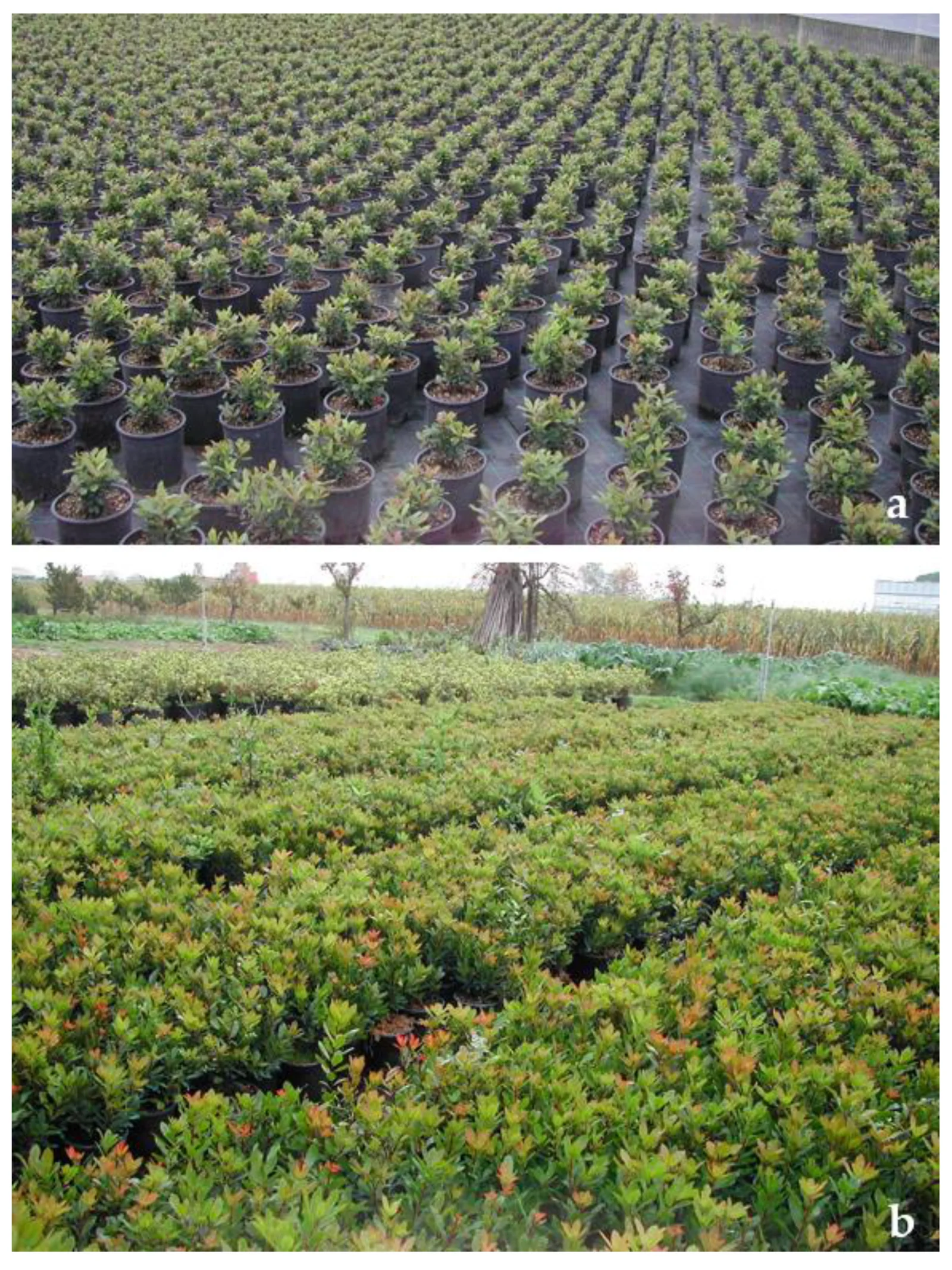
(**a**,**b**). Large-scale nursery production of potted *A. unedo* plants. Images (**a**,**b**) show seedlings at different stages of development.

The colour contrast between the evergreen foliage, the white flowers and the bright red fruit gives the plant exceptional visual impact and is one of the main reasons for its widespread use in gardens, parks and urban green spaces. Due to these characteristics, the species has become widely propagated and commercialised by the nursery industry in the Pistoia district (the European leader in ornamental plant production) in recent years, driven by the growing demand for native ornamental plants and sustainable landscaping solutions (Figure 2a,b).

Although the species is generally considered drought-tolerant, increasing climatic anomalies and more frequent extreme weather events can reduce the trees’ vigour and make them more susceptible to opportunistic pathogens. Soil-borne oomycete pathogens exploit these conditions, particularly frequent flash floods and subsequent flooding, to such an extent that they now pose an emerging threat to Mediterranean woody species. Among these, members of the genus *Phytophthora* cause root and collar diseases that can lead to crown dieback and extensive mortality in natural ecosystems, orchards, nurseries and ornamental plantations [17,18]. Their ability to survive for prolonged periods in soil through resistant propagules and to spread rapidly via motile zoospores under favourable moisture conditions makes them particularly pervasive and difficult to eradicate once established [19,20]. Consequently, *Phytophthora* species are major drivers of forest decline and biodiversity loss in natural environments and human-managed ecosystems.

One species of *Phytophthora* that is becoming a full-blown plant health emergency in the Mediterranean region is *Phytophthora cinnamomi* [21]. *A. unedo* has been reported as a host for several *Phytophthora* species but evidence of its association with *P. cinnamomi* remains limited. This oomycete was recovered in Portugal from declining *A. unedo* shrubs growing in Mediterranean maquis vegetation, which demonstrates that the species is susceptible to this pathogen under natural conditions [19]. In Italy, a *Phytophthora* isolate associated with root and collar rot in *A. unedo* had originally been identified by Scanu et al. [22] as *P. cinnamomi* var. *parvispora* Kröber & Marwitz. However, subsequent taxonomic reassessments by the same authors, based on the analyses of morphological, molecular, pathogenicity and adaptation traits, recognised *P. cinnamomi* var. *parvispora* as a distinct taxon, elevating it to the rank of species and naming it *P. parvispora* Scanu & Denman [22]. Consequently, there is currently no documented evidence of *P. cinnamomi* being present on *A. unedo* in Italy prior to the present study.

In the last few years, a decline/dieback syndrome was observed affecting *A. unedo* in a natural woodland area in Monticiano (Tuscany, central Italy), where symptomatic trees showed reduced vigour and characteristic hook-shaped shoots resulting from shoot-tip bending. Disease outbreaks were also recently recorded in some commercial nurseries producing *A. unedo* plants. In the nurseries, plants exhibited severe symptoms like leaf necrosis, extensive dark-brown necrosis of the collar and proximal roots, desiccation and mortality. This study aimed to identify the causal agent associated with the symptoms described above in both the woodland and the nurseries. Following morphological and molecular analyses, as well as pathogenicity tests, we identified the causative agent as *P. cinnamomi*. The findings are discussed in relation to the potential threat that *P. cinnamomi* poses to *A. unedo* in its natural habitat, as well as to the nursery industry.

## 2. Materials and Methods

### 2.1. Field Survey and Sampling

Etiological investigations were conducted in spring 2025 and 2026 in Tuscany, central Italy, in a natural woodland area located near Monticiano (Siena province), and in two nurseries situated in the Pistoia nursery district, on the left-hand side of the A11 motorway, along the section between Florence and Pistoia, near the outlying districts of Bonelle and Bottegone, in the municipality of Pistoia. *A. unedo* is, in fact, a species commonly found in natural areas of Mediterranean scrub and in the undergrowth of coniferous and deciduous forests, and is also widely cultivated as an ornamental plant in commercial nurseries in the Pistoia district. In the natural woodland, the survey was conducted in two consecutive years using a two-stage approach. During the first survey in spring (May–mid July) 2025, symptomatic *A. unedo* trees, between 1.5 and 2.5 m in height, of indeterminate age, were identified and a preliminary set of samples was collected from randomly selected symptomatic and asymptomatic individuals. In spring (May–June) 2026, a systematic survey was conducted within the same woodland area, during which all *A. unedo* plants were inspected for disease symptoms. In the nurseries, the survey was initiated following reports of symptomatic *A. unedo* plants. Symptomatic plants aged 1 and 2 years were identified during nursery inspections. Samples were subsequently collected from a randomly selected subset of these plants. In both environments, *A. unedo* plants exhibited dieback characterized by leaf chlorosis, progressive wilting, crown dieback, root rot, and mortality. After removing the bark at the collar, dark brown necrotic lesions extending to the proximal root system were consistently observed, suggesting the involvement of a root-borne pathogen such as a species of *Phytophthora*. In the woodland, 42 *A. unedo* trees were sampled, including 36 symptomatic and 6 apparently asymptomatic individuals. In the nurseries, 48 plants were sampled at random from a group of 107 symptomatic plants. Rhizosphere soil samples, including fine roots, were collected from a subset of symptomatic plants (16 in the woodland and 22 in the nurseries) after removing the upper organic soil layer (in the nursery, the top 2–3 cm of organic material in the pot). Four soil cores were collected around each plant, approximately 5–20 cm from the collar and bulked into a composite sample (approximately 500 mL). All fine roots showing necrotic symptoms were collected and analyzed, resulting in a total of 40 fine-root samples from woodland plants and 50 from nursery plants; in some cases, multiple necrotic roots were analyzed from the same plant. Tissue and soil samples were placed in sterile plastic bags, transported to the laboratory under refrigerated conditions, and processed within 24 h.

### 2.2. Isolation and Morphological Characterization

Root and collar tissues were washed thoroughly under running tap water, surface-sterilized in 1% NaClO for 2 min, immersed in 70% ethanol for 30 s, rinsed twice with sterile distilled water, dried on sterile filter paper, and plated onto selective PARPNH agar medium after excising small tissue fragments from the margins of necrotic lesions [23]. Plates were incubated in the dark at 24 ± 1 °C, and pure cultures were obtained by transferring individual hyphal tips onto carrot agar (CA). For *Phytophthora* isolation from soil, a baiting technique was used: aliquots of 200 mL of soil were submerged in 800 mL of distilled water in plastic containers and baited with fresh, healthy leaves of *Quercus ilex* L., *Q. suber* L., and *Viburnum tinus* L. Necrotic lesions developed on the bait leaves 2–3 days after baiting. The symptomatic leaf tissues were aseptically excised from the margins of the necrotic lesions and transferred onto V8-PARPNH selective medium for isolation. Colony morphology was assessed after growth on CA, PDA and MEA. Morphological identification was performed by examining reproductive structures, including sporangia and chlamydospores, using a light microscope. For each representative isolate, sporangial morphology (*n* = 50) and the ability to produce chlamydospores were documented. Morphometric data were obtained by measuring 50 structures per isolate at 400× magnification. The sexual compatibility of the *Phytophthora* isolates obtained from the study areas was assessed by pairing each isolate with known A1 and A2 mating type strains of *P. cryptogea* maintained in our culture collection [24]. Mycelial plugs (5 mm in diameter) from the test isolates and the reference strains were positioned approximately 4 cm apart on 10% clarified V8 agar medium. Dual cultures were incubated in darkness at 24 °C for 10 days or until oospore formation was observed. Plates were then examined for the production of oogonia and oospores in the interaction zone, and the mating type of each isolate was determined according to the pairing that resulted in sexual structure formation.

### 2.3. Molecular Identification

Genomic DNA was extracted from actively growing mycelium grown on carrot agar (CA).DNA quality and concentration were assessed spectrophotometrically, and the extracts were stored at −20 °C until use. The internal transcribed spacer (ITS) region of the ribosomal DNA and a portion of the β-tubulin (*Btub*) gene were amplified by PCR using the forward primer ITS6 (5′ GAAGGTGAAGTCGTAACAAGG 3′) and the reverse primer ITS4 (5′ TCCTCCGCTTATTGATATGC 3′) [25,26] and Btub F1 (5′ GCCAAGTTCTGGGAGGTCATC) BTubR1 (5′ CCTGGTACTGCTGGTACTCAG) [27,28], respectively. PCR reactions and amplification conditions followed those described by Benigno et al. [29]. Amplification products were purified using the NucleoSpin^®^ Gel and PCR Clean-up kit (Macherey-Nagel, Düren, Germany) according to the manufacturer’s instructions and sequenced in both directions. Forward and reverse sequences were assembled and edited to obtain consensus sequences, which were compared with those available in the NCBI GenBank database using BLASTn (http://blast.ncbi.nlm.nih.gov) to support species identification. Representative sequences were deposited in GenBank (Table 1). Sequences were aligned with ClustalX v. 1.83 [30], using the parameters reported by Bregant et al. [31]: pairwise alignment parameters (gap opening = 10, gap extension = 0.1) and multiple alignment parameters (gap opening = 10, gap extension = 0.2, transition weight = 0.5, delay divergent sequences = 25%). Alignments were checked and edited using BioEdit Alignment Editor v. 7.2.5. Individual gene phylogenies showed no major topological conflicts, supporting the concatenation of the two loci. Phylogenetic relationships were inferred using the Maximum Likelihood (ML) method implemented in MEGA-12 software [32], with the General Time Reversible (GTR) nucleotide substitution model and a gamma distribution with invariant sites (G+I), using five discrete gamma categories. All sites were included in the analysis. The initial tree was generated automatically by MEGA-12, and node support was assessed using 1000 bootstrap replicates. *Phytopythium helicoides* CBS 286.31 was used as the outgroup to root the tree.

### 2.4. Pathogenicity Test

Pathogenicity tests were conducted using two *P. cinnamomi* isolates (PCV03 and PCM11) on two-year-old *A. unedo* plants maintained under controlled environmental conditions. Twenty plants were inoculated with each isolate, while an additional 20 plants inoculated with sterile carrot agar (CA) plugs; sterile pond water served as controls. For each inoculated plant, a combined collar and soil inoculation approach was used. The inoculated region of the collar was first surface-disinfected with 70% ethanol, and a small piece of outer and inner bark (5 mm in diameter) was removed using a flamed cork borer. The excised tissue was replaced with a 5-mm-diameter agar–mycelium plug excised from the margin of an actively growing colony on CA. The inoculation site was covered with cotton wool soaked in sterile water and wrapped in aluminium foil. At the same time, the potting substrate was infested with 100 mL of a zoospore suspension (2 × 10^5^ zoospores mL^−1^). The zoospore suspension was prepared from 7-day-old cultures grown on CA and flooded with sterile pond water for 24 h to induce sporangial formation and zoospore release. Control plants received a sterile CA plug and 100 mL of sterile pond water. The plants were maintained in a growth chamber at 25 °C and watered regularly throughout the experiment. Disease development was monitored regularly for 30 days. At the end of the experiment, all plants were carefully removed from their pots, and disease severity was assessed by examining the development of collar necrosis and root rot. *P. cinnamomi* was re-isolated from symptomatic collar and root tissues using the isolation procedure described above to fulfil Koch’s postulates. The identity of the recovered isolates was confirmed based on colony morphology, micromorphological characteristics, and sequencing of the ITS region.

## 3. Results

### 3.1. Field Surveys

**Figure 3:**
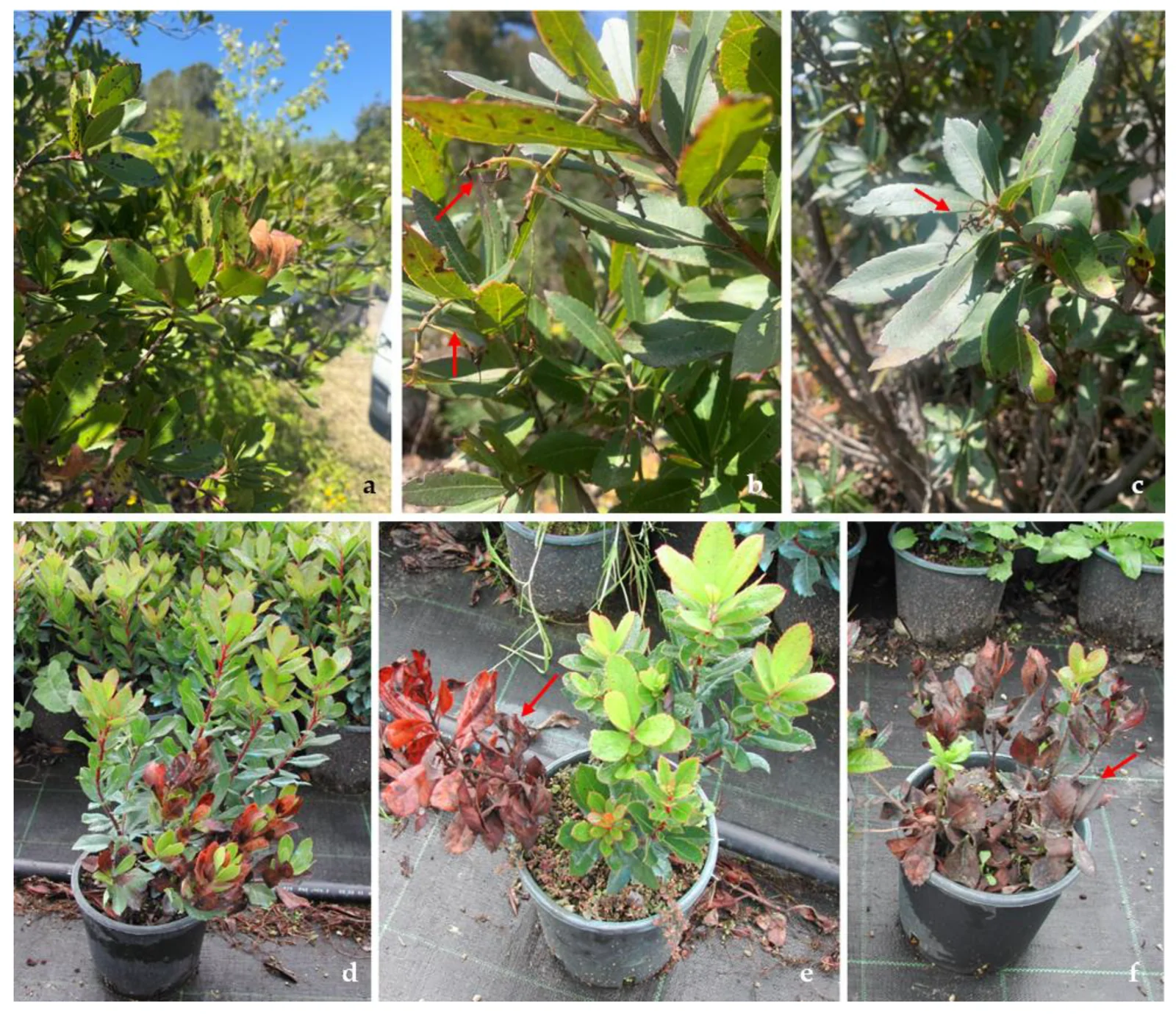
Symptoms associated with root and collar rot in naturally affected woodland and nursery-grown *Arbutus unedo* plants. (**a**–**c**) Under natural conditions, infected plants showed a marked reduction in vegetative vigour, as well as the presence of deformed apical shoots with a characteristic curved (‘hooked’) shape. (**d**–**f**) Nursery-grown plants showing symptoms of varying severity. Arrows indicate characteristic hooked shoot tips and necrotic twigs.

In the woodland, *A. unedo* trees generally exhibited reduced vigour and stunted growth. These symptoms were associated to shoots with a bent, hook-shaped apex. In some trees, these tips subsequently withered (Figure 3a–c). Inspection of the root collar revealed brownish necrotic lesions beneath the bark, extending from the basal stem towards the proximal roots. Root systems of symptomatic individuals also showed signs of necrosis. These symptoms were observed in only 36 of the 42 trees sampled (36 symptomatic and 6 asymptomatic).

In the nurseries, plant exhibited generally more severe symptoms, ranging from leaf chlorosis and yellowing to partial canopy dieback, followed by the desiccation of branches and shoots, and ultimately complete crown mortality (Figure 3d–f). A total of 107 symptomatic plants were recorded during the survey. Of these, 68% exhibited partial canopy dieback and 32% were dead.

### 3.2. Isolation and Identification of Phytophthora cinnamomi

A total of 184 isolates of *P. cinnamomi* were recovered from root collar tissues, fine roots and rhizosphere soil of 84 symptomatic plants (36 from the woodland and 48 from the nurseries). Of these, 69 were obtained from naturally infected trees in the woodland. No *P. cinnamomi* isolates were recovered from the six apparently symptom-free plants sampled. A total of 115 isolates were obtained from tissue samples taken from basal stems, fine roots, and from the rhizosphere soil of plants grown in the nurseries (Table 1).

**Figure 4:**
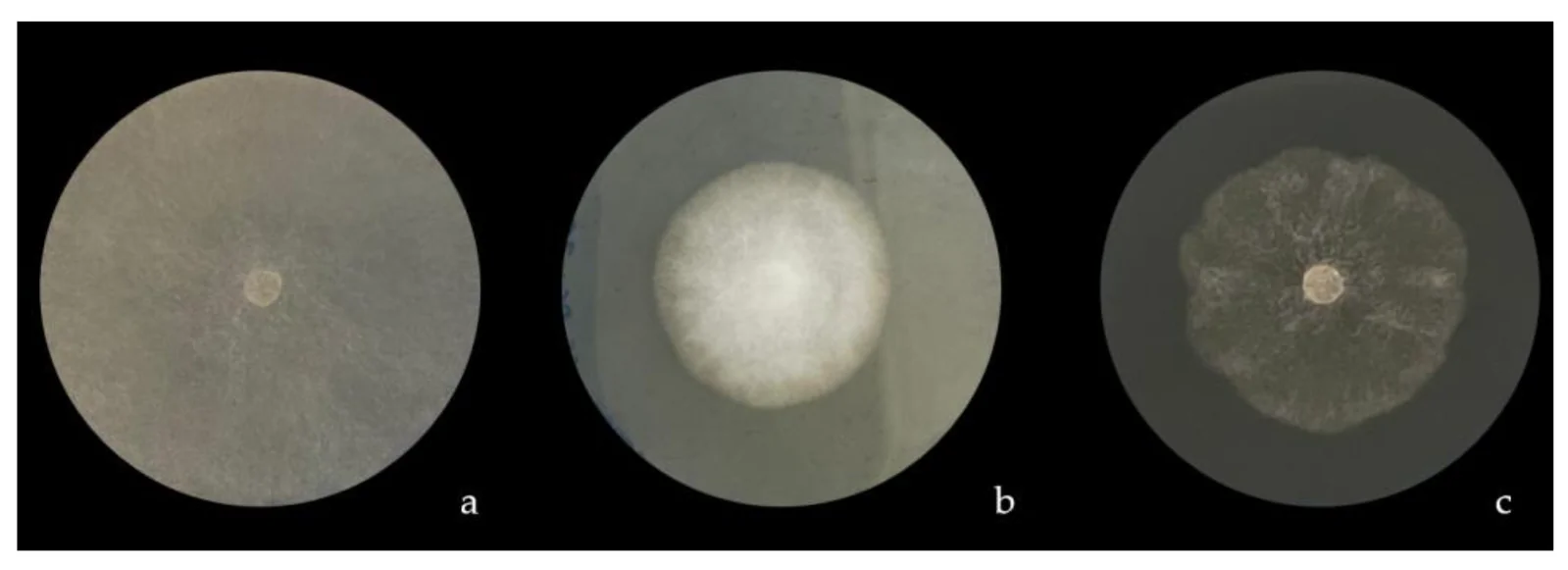
Cultural characteristics of the PCV03 isolate, selected as a representative isolate since all strains examined in the study exhibited comparable macroscopic features across the different growth substrates. Colonies showed a consistent morphological pattern, characterised by abundant aerial mycelium production and regular growth on CA (**a**) and MEA (**b**). In contrast, colonies grown on PDA (**c**) displayed the typical rosette (petaloid) growth pattern.

The macro- and micromorphological characteristics of all isolates were examined. Isolates showing comparable colony morphology and micromorphological features were assigned to the same morphotype, thereby allowing isolates with consistent phenotypic characteristics to be grouped for subsequent identification and analyses. Colonies developed an abundant aerial mycelium with a uniform growth pattern on CA (Figure 4a) and MEA (Figure 4b), and a rosette (petaloid) pattern on PDA (Figure 4c).

All isolates consistently produced an abundance of chlamydospores in CA medium. Based on these shared morphological features and subsequent molecular analyses, all isolates were identified as *P. cinnamomi*. Colonies grown on carrot agar (CA) developed abundant aerial mycelium. The isolates produced non-papillate, persistent sporangia with internal, occasionally nested, proliferation. Sporangia were predominantly ovoid, ellipsoid or obpyriform, and only rarely irregular in shape, measuring 12–78 × 11.5–42 μm. They were borne on unbranched or simple sympodial sporangiophores. Hyphal swellings were abundant, globose to subglobose or coralloid, frequently occurring in clusters or aggregates. Chlamydospores were abundant, globose, terminal or intercalary, and only occasionally lateral, measuring 16.6–42 μm in diameter.

Pairing tests with reference A1 and A2 mating-type strains demonstrated that all isolates were heterothallic and belonged to the A2 mating type. Sexual structures were produced only in pairings with the A1 tester strain. Oogonia were smooth-walled and spherical, measuring 22–45 μm in diameter, with amphigynous, spherical to broadly ellipsoid antheridia. Oospores were predominantly plerotic, with moderately thick walls, and measured 20–34 μm in diameter.

All 184 isolates had morphological features corresponding to the selected morphotypes, and the molecular identification of six representative isolates confirmed that they all belonged to *P. cinnamomi*. Sequencing of the ITS region and the β-tubulin gene confirmed that all six isolates belonged to *P. cinnamomi*. The remaining isolates exhibited macro- and micromorphological traits that matched each of the six morphotypes, and were therefore assigned to the relevant morphotype. Sequences obtained from isolates collected in the woodland and nurseries were identical (Table 2). NCBI BLAST analyses revealed 100% sequence identity with reference sequences of *P. cinnamomi* deposited in GenBank.

**Figure 5:**
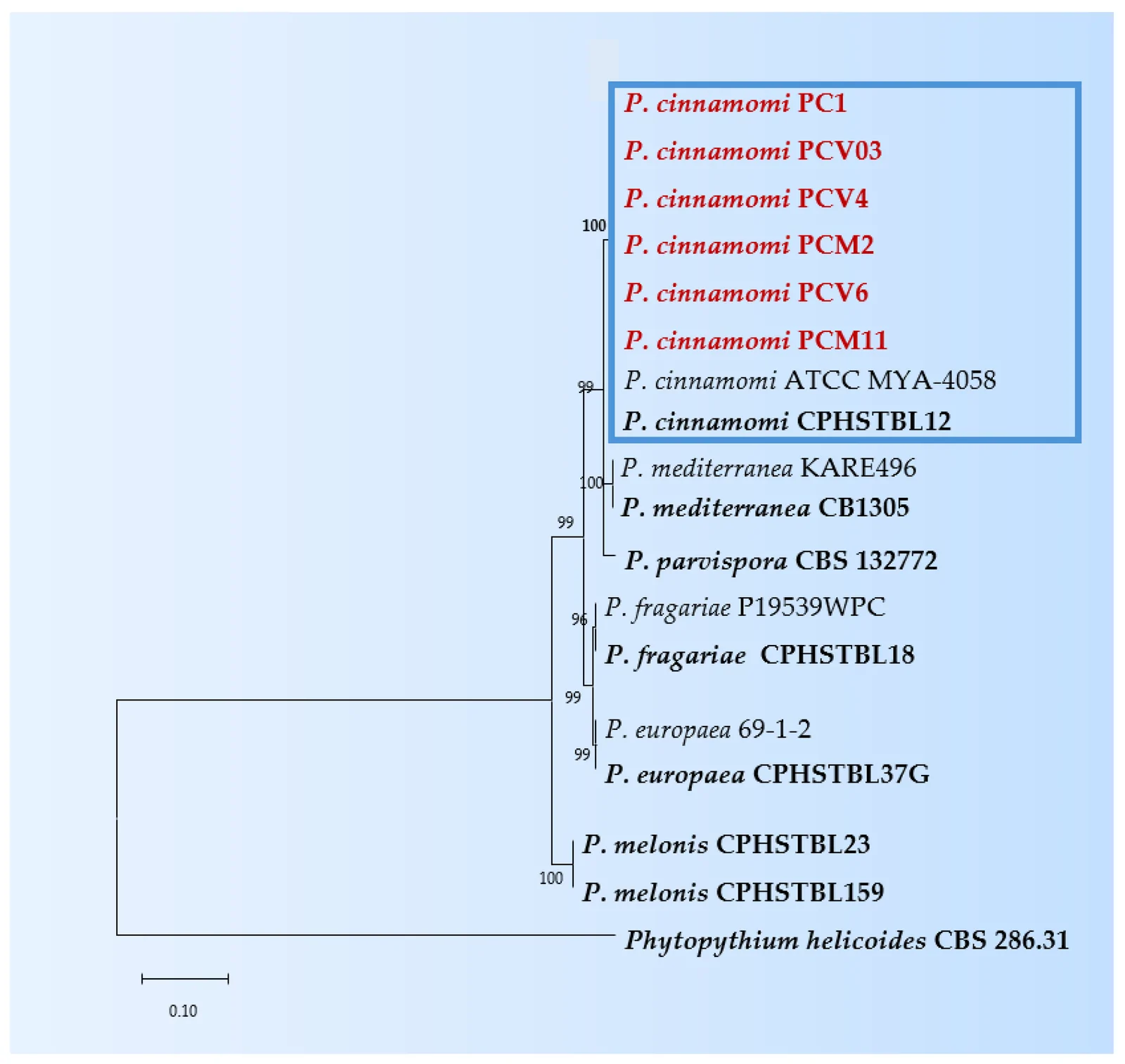
Maximum-likelihood phylogenetic tree based on concatenated ITS and β-tubulin (*β-tub*) sequences (1825 bp). *Phytopythium helicoides* CBS 286.31 was used as the outgroup. Bootstrap values from 1000 replicates are indicated at the nodes. Isolates from the present study are shown in red and enclosed in the blue box, along with the *P. cinnamomi* ex-type. The scale bar indicates 0.10 substitutions per site.

The phylogenetic analysis was based on a concatenated dataset comprising ITS and β-tubulin (β-tub) sequences, yielding a total alignment length of approximately 1825 bp. The combined dataset comprised an 850 bp ITS fragment and a 975 bp β-tubulin fragment. Of the 123 parsimony-informative sites identified across the combined alignment, 60 were located within the ITS region and 63 within the β-tubulin locus. All isolates obtained in the present study clustered within *Phytophthora* clade 7, together with the ex-type and reference isolates of *P. cinnamomi*, with strong bootstrap support (ML bootstrap = 100%). *Phytopythium helicoides* CBS 286.31 was used as the outgroup. Bootstrap values based on 1000 replicates are indicated at the nodes (Figure 5).

### 3.3. Pathogenicity Tests

**Figure 6:**
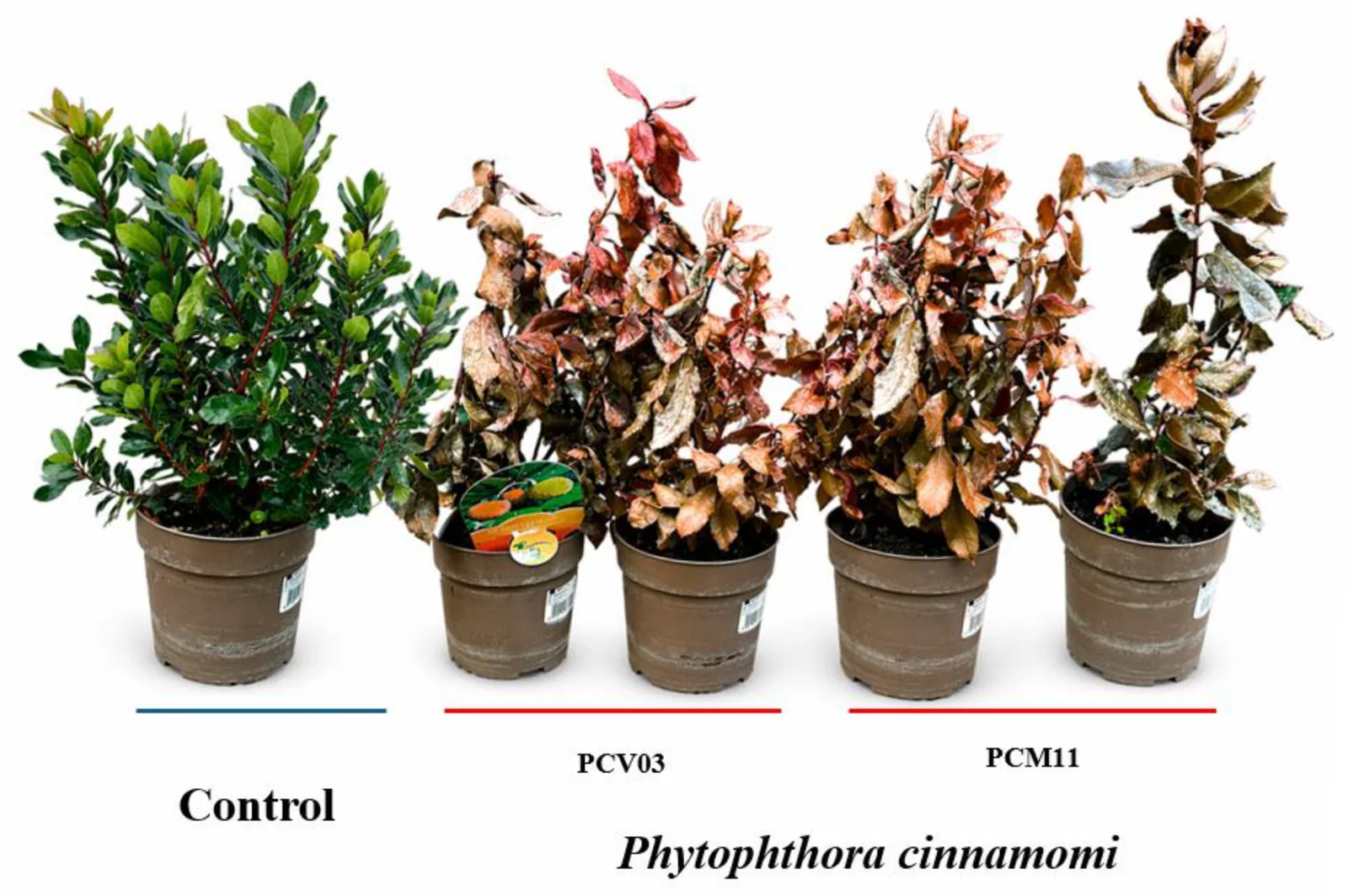
Pathogenicity test on two-year-old *Arbutus unedo* plants. On the left: uninoculated (control) plants, treated with sterile agar and water, exhibiting normal growth and healthy foliage. From left to right (4 pots): plants inoculated with *P. cinnamomi* (isolates PCV03 and PCM11). These plants showed root rot and a progressive dieback, with leaf yellowing and wilting, resulting in total necrosis of the crown and death. The severity of these symptoms varied between the control and the treatments.

Both *Phytophthora cinnamomi* isolates (PCV03 and PCM11) induced disease in all inoculated *A. unedo* plants. The first symptoms appeared approximately 20 days after inoculation and consisted of leaf chlorosis, loss of turgor, and progressive wilting. As disease progressed, plants developed extensive root rot, dark brown necrotic lesions at the root collar extending into the proximal root system, and severe canopy dieback. All inoculated plants (100%) developed extensive necroses and died within 30 days; no mortality was observed in the control group. No difference in virulence was observed among the two isolates. As all inoculated plants showed complete necrosis of their aerial parts, it was not possible to detect quantitative differences in disease severity between the two isolates. Control plants remained healthy and symptomless throughout the experimental period, showing no evidence of root rot, collar necrosis, or canopy decline (Figure 6). *P. cinnamomi* was consistently re-isolated from the collar and root tissues of all symptomatic, inoculated plants. The identity of the reisolated strains was confirmed through an analysis of their macro- and micro-morphological traits. The pathogen was re-isolated from all 40 inoculated plants (20 per treatment). As all cultures were morphologically identical, one representative isolate per treatment was re-identified by ITS and β-tubulin sequence analysis. No *Phytophthora* isolate was recovered from control plants. These results fulfilled Koch’s postulates and confirmed *P. cinnamomi* as the causal agent of collar rot, root rot, and decline of *A. unedo*.

## 4. Discussion

The present study provides the first confirmed evidence that *Phytophthora cinnamomi* is associated with root and collar rot of *Arbutus unedo* in Italy and is responsible for the symptoms observed in the above-ground and below-ground parts of plants in the woodland and in nurseries. The pathogen, consistently recovered from symptomatic root, collar tissues and rhizosphere was unequivocally identified by coupling morphological and molecular methods.

The pathogenicity assay confirmed the ability of *P. cinnamomi* to induce severe disease in *A. unedo* under the combined collar and soil inoculation conditions. Both isolates caused severe disease, and the pathogen was successfully re-isolated in pure culture from inoculated plants, confirming the pathogenic role of *P. cinnamomi* and fulfilling Koch’s postulates. The dual inoculation procedures applied to the same plants, however, did not allow to determine the relative contribution of each route of infection to symptom development. Although the hook-shaped shoot-tip symptom was not specifically reproduced in the pathogenicity assay, its occurrence in symptomatic plants and absence in asymptomatic ones, combined with the negative results of *P. cinnamomi* isolation from this latter group, suggests an association, even though the causal relationship remains unconfirmed. *P. cinnamomi* is considered one of the world’s most damaging soil-borne plant pathogens, owing to its high virulence, exceptional host breadth, persistence in soil, and widespread distribution across continents and ecosystems [33,34,35,36]. Current estimates indicate that its host range encompasses more than 5000 plant species, including trees, shrubs, ornamentals, and major crop hosts [37,38,39]. Compared with the roughly 950 host species recognized in earlier compilations, this sharp increase in documented susceptibility underscores how advances in surveillance and pathogen detection have expanded understanding of its host range [20,40].

In susceptible hosts, *P. cinnamomi* causes a range of symptoms, including the destruction of fine roots, severe root rot and necrosis, and disruption to water and nutrient uptake. These symptoms can ultimately lead to defoliation, loss of vigour, and eventual mortality [41,42,43].

In the Mediterranean area, this pathogen is widely recognised as a significant biotic factor contributing to the decline/dieback of holm oak and cork oak forests as well as dehesas [44,45,46]. Several field studies conducted in Portugal and southern Italy have repeatedly detected the pathogen in declining stands, with a higher recovery rate from declining trees in comparison to healthy trees, and a robust association with host mortality [21,47,48].

The impact of climate on disease transmission is multifaceted, with infections being favoured by moist soils and warm conditions [41,43,49]. However, extreme rainfall can also boost inoculum and disease spread, even if drought alone does not necessarily predispose infection. It has been demonstrated that a variety of coexisting woody, shrubby or herbaceous species can act as reservoir hosts, thereby maintaining the pathogen in the soil. This phenomenon contributes to the understanding of the persistence of the pathogen and the chronic suffering of Mediterranean shrublands and forests [21,50,51]. Moreover, climate change is expected to further enhance its host range and distribution as well as the severity of its attacks by creating environmental conditions more favourable to its survival and dispersal. These epidemiological implications are closely linked to the pathogen’s ability to persist in the environment through a range of survival mechanisms. *P. cinnamomi* can persist in soil and infected plant tissues through a range of survival structures, including chlamydospores, oospores, stromata, and hyphal aggregates within lignitubers [37]. Chlamydospores have been demonstrated to facilitate short-to-medium-term survival between consecutive rain events and in moderately dry conditions [52,53]. However, they are unlikely to serve as the primary long-term survival structure, as free chlamydospores in soil have been shown to lose viability within 12 weeks [52]. The long-term persistence of the oomycete, particularly during the hot and dry Mediterranean summers, is instead achieved through selfed oospores, stromata, and hyphae in infected root tissue of both host and non-host species [52,54,55]. Mycelial growth in infected tissues has been demonstrated to enable short-term persistence and can sustain the pathogen biotrophically in asymptomatic hosts [37,55,56]. In favourable moisture conditions, the pathogen produces motile, biflagellate zoospores, which are the primary infective agents and promote local dispersal through the movement of water [38].

The discovery of *P. cinnamomi* on *A. unedo* in natural environments is particularly significant given the ecological importance of this host in Mediterranean forests and shrublands. As a key component of these ecosystems, *A. unedo* performs multiple ecological functions and provides a wide range of ecosystem services, thereby contributing to biodiversity conservation, ecosystem stability, and resilience. Its ability to thrive in urban environments, combined with its ornamental value, has also contributed to its widespread use and commercial success in the nursery trade. However, the occurrence of *P. cinnamomi* on such a widely marketed host raises important phytosanitary concerns, particularly regarding the potential movement of the pathogen through infected, asymptomatic nursery stock, and its subsequent establishment in urban and natural environments. Consequently, the production and commercialization of pathogen-free planting material, supported by effective surveillance and appropriate nursery hygiene measures, may help reduce the potential risk of pathogen dissemination [18,57].

Interestingly, despite the susceptibility of *A. unedo* to *P. cinnamomi*, several studies have shown that this plant produces secondary metabolites with anti-oomycete activity [58]. Both arbutin and its hydrolysis product, hydroquinone, significantly inhibited the mycelial growth of *P. cinnamomi* in vitro, with reductions approaching 80% at the highest concentrations tested. Methanolic extracts of *A. unedo* leaves also exhibited strong anti-oomycete activity in microplate assays, with a minimum inhibitory concentration corresponding to 0.625 mg mL^−1^ of arbutin, substantially lower than that recorded for purified arbutin alone (12 mg mL^−1^) [58]. These findings suggest that synergistic interactions among multiple metabolites in the leaf extracts may account for their enhanced inhibitory activity. Nevertheless, there is currently no evidence that these compounds provide effective protection against *P. cinnamomi* under field conditions or during natural host–pathogen interactions. This is supported by the disease symptoms observed in naturally infected plants in both forest and nursery environments, as well as in experimentally inoculated plants.

## 5. Conclusions

*P. cinnamomi* has been shown, in a variety of contexts, to be capable of altering the composition and structure of plant communities, reducing biodiversity and compromising the resilience of ecosystems. Future phytosanitary and monitoring efforts should aim to assess and clarify the environmental factors influencing infection and disease development as well as the direction of transmission between natural communities and nurseries. Such knowledge would be essential for the development of effective surveillance and management strategies aimed at preventing the further spread of this destructive soil-borne pathogen.

## Figures and Tables

**Table 1:** Isolation frequency of *Phytophthora cinnamomi* from collar tissues, rhizosphere soil, and fine roots of *Arbutus unedo* sampled in woodland and nursery settings.

Setting	Sample Type	Positive Samples/Samples Examined (%)
Woodland	Collar tissues	27/36 (75.0%)
Woodland	Rhizosphere soil	16/16 (100%)
Woodland	Fine roots	26/40 (65%)
Nursery	Collar tissues	44/48 (91.7%)
Nursery	Rhizosphere soil	22/22 (100%)
Nursery	Fine roots	49/50 (98.0%)
Total		184/212 (86.79%)

**Table 2:** *Phytophthora cinnamomi* isolates obtained in the present study, including isolate codes, origin, and GenBank accession numbers for the ITS and β-tubulin (*β-tub*) sequences.

Isolate Code	Taxon	Origin	Year	Source Material	ITS	β-Tub
PC1	*P. cinnamomi*	Woodland	2025	Rhizosphere soil	PZ671180	PZ671987
PCV03	*P. cinnamomi*	Nursery	2026	Rhizosphere soil	PZ671183	PZ671988
PCV4	*P. cinnamomi*	Nursery	2026	Fine roots	PZ671182	PZ671989
PCM2	*P. cinnamomi*	Nursery	2026	Collar tissue	PZ671181	PZ671990
PCV6	*P. cinnamomi*	Woodland	2026	Collar tissue	PZ626853	PZ661498
PCM11	*P. cinnamomi*	Woodland	2026	Fine roots	PZ631085	PZ661499

## Data Availability

The original contributions presented in this study are included in the article. Further inquiries can be directed to the corresponding author.
